# Sexual Masochism Disorder with Asphyxiophilia: A Deadly yet Underrecognized Disease

**DOI:** 10.1155/2016/5474862

**Published:** 2016-09-22

**Authors:** Anna Coluccia, Mario Gabbrielli, Giacomo Gualtieri, Fabio Ferretti, Andrea Pozza, Andrea Fagiolini

**Affiliations:** ^1^Department of Medical Sciences, Surgery and Neurosciences, Santa Maria alle Scotte University Hospital of Siena, Siena, Italy; ^2^Department of Molecular Medicine, University of Siena School of Medicine, and Department of Mental Health, University of Siena Medical Center (AOUS), Siena, Italy

## Abstract

DSM-5 distinguishes between paraphilias and paraphilic disorders. Paraphilias are defined as atypical, yet not necessarily disordered, sexual practices. Paraphilic disorders are instead diseases, which include distress, impairment in functioning, or entail risk of harm one's self or others. Hence, DSM-5 new approach to paraphilias demedicalizes and destigmatizes unusual sexual behaviors, provided they are not distressing or detrimental to self or others. Asphyxiophilia, a dangerous and potentially deadly form of sexual masochism involving sexual arousal by oxygen deprivation, are clearly described as disorders. Although autoerotic asphyxia has been associated with estimated mortality rates ranging from 250 to 1000 deaths per year in the United States, in Italy, knowledge on this condition is very poor. Episodes of death caused by autoerotic asphyxia seem to be underestimated because it often can be confounded with suicide cases, particularly in the Italian context where family members of the victim often try to disguise autoerotic behaviors of the victims. The current paper provides a review on sexual masochism disorder with asphyxiophilia and discusses one specific case as an example to examine those conditions that may or may not influence the likelihood that death from autoerotic asphyxia be erroneously reported as suicide or accidental injury.

## 1. Introduction

Sexual masochism disorder (SMD) has been reported in a percentage ranging from 1 to 5% of the US and Australian general population [1]. In a Canadian study, sadomasochistic sexual fantasies during sexual intercourse were reported by 10% of men and a large percentage of females (from 31 to 57%) were reported to have rape fantasies [2]. In a survey of sexual behavior in the US involving 2026 respondents, Hunt [3] found that 4.8% of males and 2.1% of females reported ever having obtained sexual pleasure from inflicting pain and 2.5% of males and 4.6% of females from receiving pain. A review [4] of the literature on women's rape fantasies reported that 31–57% of women had fantasies in which they were forced into sex against their will and that for 9–17% these were a frequent or favorite fantasy experience.

Asphyxiophilia is one of the most dangerous conditions associated to SMD and is characterized by the use of various strategies to achieve the level of oxygen depletion needed to enhance sexual arousal, such as self-strangulation, hanging, suffocation with an object like a plastic bag over the head, chest compression, use of gas or volatile solvents, or a combination of these, up to the point of loss of consciousness [5]. Participants often establish some sort of “rescue mechanism” as a safety release in case they lose consciousness. Sometimes, however, this safety release does not function correctly or the individual incorrectly measures the amount of oxygen restriction, which may lead to death or serious brain injury.

Estimates of the mortality rate of autoerotic asphyxia range from 250 to 1000 deaths per year in the United States [6]. In a review of all published cases of autoerotic deaths from 1954 to 2004, Hucker [7] reported that autoerotic death practitioners were predominantly Caucasian males. Most cases of asphyxia were by hanging, ligature, plastic bags, chemical substances, or a mixture of these. Atypical methods of autoerotic activity leading to death accounted for about 10.3% of cases in the literature and included electrocution (3.7%), overdressing/body wrapping (1.5%), foreign body insertion (1.2%), and atypical asphyxia method (2.9%). Victims were aged from 9 to 77 years and were mainly found in various indoor locations [7].

Because of its dangerousness, the DSM-5 paraphilias workgroup decided that this condition merits a specifier and patients be diagnosed with SMD with asphyxiophilia. Studies of asphyxiophilia survivors indicate that nearly all individuals fantasize about masochistic scenarios as they engage in asphyxia practices [8].

Asphyxiophilia may be accomplished by a self-induced or assisted cerebral anoxia, usually by hanging, suffocation, or reduction of the oxygen in the inspired air that may be achieved through plastic bags or gas masks that may allow inhaling some anesthetic gases (e.g., chloroform and nitrous oxide) and volatile chemicals (e.g., isopropyl nitrite and isobutyl nitrite), by compressing the chest, or chemically through the use of narcotics, usually but not always during masturbation or some other sexual activity [9, 10]. Self-hanging appears the most common method observed among fatal cases [10].

Asphyxiophilia can be often accompanied with other paraphilias such as bondage and transvestism and a great range of paraphernalia (props and devices involved in paraphilia sexual activities), sexual aids or pain-stimulating agents, intimate feminine garments, bondage, locks, pornographic magazines, and rubber items [11]. The inhalation of anesthetics, inhalants, and solvents sometimes occurs in combination with other appropriate devices like gas masks, anesthetic masks, diving masks, or even anesthetic machines [12]. The individual may inhale the substances or more often soak a rag with a solvent and then insert the rag in the mouth to inhale the fumes. Jones and colleagues [13] described a case of asphyxiophilia characterized by plastic-bag asphyxiation in combination with inhaled glue spray. Similarly, Gowitt and Hanzlick [14] reported two cases which had an involvement of l-1-l-trichloroethane, a compound commonly found in typewriter correction fluid.

Fatal masochistic asphyxia tends to be a relatively rare phenomenon and may be caused by the malfunction of apparatus used to provide sexual pleasure. Asphyxiation due to neck compression subsequent to hanging has been reported as the most common form of autoerotic death [15]. However, differentiation between accidental death and suicide often in cases of suspicion of autoerotic asphyxia becomes difficult due to the unusual methods used by victims [15]. According to Arun et al. [16], between 5 and 20 percent of deaths across the US that need to be certified are considered equivocal, but most of them are undetermined between accident and suicide. The possibility of homicide should be ruled out in the cases of unusual suicides as well [16]. The psychological autopsy has been generally accepted in the psychiatric field for evaluating suicide cases for better understanding the social, psychological, and physical conditions of suicide [17]. Establishing whether the death was accidental or suicidal is quite challenging for the forensic professionals. Some authors established criteria of accidental autoerotic death: solitary, accidental, and caused directly by the abnormal mechanism aimed at sexual satisfaction [18]. Some features differentiating death attributed to autoerotic asphyxia and suicide are presented in Table 1.

Several sources in the literature, some dating back more than 200 years, reported the use of life-threatening sexual practices, particularly those related to autoerotic asphyxia [19]. Recently, increasing evidence has been described by authors on this topic. Ueno and colleagues [20] reported the case of sexual asphyxial death by of a 35-year-old male, found dead lying on his face in a bed of a truck cab and hanging himself from a window frame using a leather belt. He was completely naked. There were pornographic and sadomasochistic magazines beneath his face, opened to pages depicting nude photographs of a woman. Autopsy findings revealed a ligature mark on the neck and petechial hemorrhages in the conjunctivae, but there were no hemorrhages in the neck muscles or fractures of the hyoid bone or the thyroid cartilage. The circumstances suggested that the death of the man was accidental and due to asphyxia by hanging performed to enhance sexual gratification during masturbation [20]. Some evidence also suggested that autoerotic asphyxia presents with characteristics similar to those found in scenes of male cases, despite the fact that autoerotic practices are generally believed to be rarer among females than in males. Skugarevsky et al. [21] conducted a review of cases of autoerotic asphyxia among women. The authors identified four cases, in which the autopsies showed that asphyxiation was the cause of death. In two cases death was determined by suffocation due to strangulation and in the other two by plastic bags placed over the individuals' head. The authors observed that in one case there was additional evidence at the scene that the deceased had inhaled ether.

## 2. Aims

Despite the fact that growing interest has been dedicated by researchers and practitioners for this topic, features of autoerotic asphyxia seem to be understudied in Italy. In light of the limited amount of data on cases of death caused by autoerotic asphyxia in the Italian context, the current study described the characteristics of a case of an Italian man who died due to suffocation during an autoerotic practice. Elements of the scene were described in order to provide a rationale for considering this case as a death due to autoerotic asphyxia.

## 3. Case Report

A male aged 47 years and married but without children was found dead in his house. The corpse was found by the neighbors near the table in the cellar. The neighbors were alerted by the wife of the man, who had not seen him going back home for dinner. A slipknot was around the neck and the cord was attached to the ceiling beam. The legs were flexed on the thighs and the body seemed to be on his knees, but the knees were suspended a few inches from the floor. The legs were also suspended and crossed, while the feet touched the floor. The hanging was therefore incomplete since the body was not completely suspended from the ground. The man was wearing sporty clothing, white T-shirt with short sleeves, white shorts, cotton socks, sneakers, and pantyhose on the head as a hood.

External examination of the corpse, stripped of clothing, evidenced the absence of the underwear. After the pantyhose and the hood were removed, the presence of a discontinuous groove on the neck was found, consistent with the death due to hanging. No external cause injury was observed. An acute mechanical asphyxiation was identified as the cause of death.

Reconstructing the scene based on the evidence and the data obtained seemed to support the hypothesis that the subject had achieved sexual pleasure through autoerotic asphyxiation. A suicide or another cause of accidental death was excluded based on the way the man was dressed.

## 4. Discussion

The circumstantial evidence demonstrated that the death occurred while the subject was enacting autoerotic maneuvers. The literature shows the noticeable diffusion of particular methods to reach sexual gratification but the incidents of such practices are underestimated because the cases are reported as suicides. In the book Autoerotic Fatalities published in 1983 by the FBI Agent Hazelwood, Psychiatrist Park Dietz and Dr. Ann Burgess [22] delineated the criteria to correctly diagnose autoerotic accidental death (AAD): (1) the asphyxiation being caused by strangling or hanging, body positioning that favors asphyxiation as the cause of death, and the asphyxiation death being accidental; (2) evidence at the crime scene proving that the lifesaving system failed; (3) proof that the sexual activity was solitary (in other cases it would be characterized as homicide or assisted suicide); (4) proof of sexual fantasy materials such as pornography; (5) proof of previous acts of autoerotic asphyxiation; and (6) missing intent of an apparent suicide. With regard to the current case, (1) death was caused by mechanical asphyxia acute action of a noose tied around the neck; (2) the suspension of the body was less than the height of the subject that flexing the legs could voluntarily and independently cause the progressive and gradual tensioning and fastening tightening the noose tied around the neck, checking asphyxia exerted by the loop itself. Conversely, the extension of the lower limbs would have allowed reducing asphyxia, representing a mechanism of self-protection, but hypoxia has generated a loss of consciousness and muscle relaxation, which prevented maintaining the voluntary control of self-induced condition with the consequent lowering of the body and closing the noose around the neck. (3) The surveys and investigations excluded the presence of the investigators at the time of the offense, other people in the place of death, suggesting a sexual activity alone. (4) From reading the tale, found by investigators in the job office, it emerged that the pantyhose can be an important element of the sexual fantasy of the subject. (5) In 2005 his wife had found a video-tape, in which the man had simulated a hanging similar to that in which the subject was found dead. When his wife asked him some explanations, he released vague statements and did not want to talk about it. (6) An apparent suicidal intent was missing (missing a suicide note).

The diagnosis of an AAD appeared correct, since the evidence was similar to the elements outlined in the above-mentioned literature [22] while the homicidal hypothesis was not probable (the entry points of the apartment were closed from inside and there were no signs of a third party presence), and the external examination of the corpse did not evidence signs of violence or struggles.

Moreover, evidence for an AAD seemed to be further supported since in the job office of the man the police found a tale, where he described autoerotic sexual practices similar to those related to the death.

On the basis of the familiar testimony and the hypothesis of the investigators, suicide seemed improbable. After police acquaintances the subject seemed to conduct a normal life and did not have a psychiatric case history. The subject was close to his family and did not show homosexual tendencies, and the literature does state that such practices are often enacted by largely heterosexual individuals who lead normal lives.

## 5. Conclusions

Episodes of death caused by autoerotic asphyxia seem to be underestimated to date because they often can be confounded with suicide cases, particularly in the Italian context where family members of the victim often try to disguise autoerotic behaviors of the victims. In line with these considerations, it should be highlighted that the current case was found by the neighbors. In addition, the current case presents with original features compared with previous case reports since the victim was found wearing pantyhose on the legs, and this feature could be interpreted as a fetishistic behavior based on cross-dressing. However, the lack of information on the psychological anamnesis prevented drawing this conclusion.

Moreover, the current case report highlights the importance that a correct and reliable diagnosis of autoerotic asphyxia cases is based on a multidisciplinary staff consisting of legal physicians and investigators who jointly work using the literature and their experience.

In conclusion, for cases of autoerotic death we suggest the importance of taking into account evidence and elements coming from a variety of sources, including the examination of the victim's psychological history and familiarity, since for the current case father of the victim was suicidal, and the investigation of the death context and environment.

More research and public awareness are needed as to the risks of autoerotic asphyxiation, which needs to be widely recognized as an extremely dangerous sexual practice and should not be used to achieve sexual gratification.

## Figures and Tables

**Table 1:** Comparison of the characteristics of death due to autoerotic asphyxia and suicide.

Death due to autoerotic asphyxia	Suicide
The deceased died of asphyxia due to a binding that was designed to cause hypoxia while he/she was engaged in some form of solitary sexual activity	Absence of some form of sexual activity

Participants often adopt a “rescue mechanism” in case they lose consciousness	It rarely involves rescue mechanisms

It does not involve antemortem evidence of suicidal ideation or depression	It involves antemortem evidence of suicidal ideation or depression

It can be often accompanied with other paraphilias (e.g., bondage and transvestism)	It is not accompanied with paraphilias

Predominant rates among men relative to women	Predominant rates among men relative to women
